# Arbuscular Mycorrhizal Fungi Community Structure, Abundance and Species Richness Changes in Soil by Different Levels of Heavy Metal and Metalloid Concentration

**DOI:** 10.1371/journal.pone.0128784

**Published:** 2015-06-02

**Authors:** Ramasamy Krishnamoorthy, Chang-Gi Kim, Parthiban Subramanian, Ki-Yoon Kim, Gopal Selvakumar, Tong-Min Sa

**Affiliations:** 1 Department of Environmental and Biological Chemistry, Chungbuk National University, Cheongju, Chungbuk, Republic of Korea; 2 Bio-Evaluation Center, Korea Research Institute of Bioscience and Biotechnology, Cheongwon, Republic of Korea; University of Coimbra, PORTUGAL

## Abstract

Arbuscular Mycorrhizal Fungi (AMF) play major roles in ecosystem functioning such as carbon sequestration, nutrient cycling, and plant growth promotion. It is important to know how this ecologically important soil microbial player is affected by soil abiotic factors particularly heavy metal and metalloid (HMM). The objective of this study was to understand the impact of soil HMM concentration on AMF abundance and community structure in the contaminated sites of South Korea. Soil samples were collected from the vicinity of an abandoned smelter and the samples were subjected to three complementary methods such as spore morphology, terminal restriction fragment length polymorphism (T-RFLP) and denaturing gradient gel electrophoresis (DGGE) for diversity analysis. Spore density was found to be significantly higher in highly contaminated soil compared to less contaminated soil. Spore morphological study revealed that *Glomeraceae* family was more abundant followed by *Acaulosporaceae* and *Gigasporaceae* in the vicinity of the smelter. T-RFLP and DGGE analysis confirmed the dominance of *Funneliformis mosseae* and *Rhizophagus intraradices *in all the study sites. *Claroideoglomus claroideum*, *Funneliformis caledonium*, *Rhizophagus clarus* and *Funneliformis constrictum* were found to be sensitive to high concentration of soil HMM. Richness and diversity of *Glomeraceae* family increased with significant increase in soil arsenic, cadmium and zinc concentrations. Our results revealed that the soil HMM has a vital impact on AMF community structure, especially with *Glomeraceae* family abundance, richness and diversity.

## Introduction

Huge amount of heavy metal and metalloid (HMM) are released into the soil and water by mining and smelting processes [1]. These HMM have adversely affected micro and macro organism habitats in terrestrial and aquatic ecosystems [1,2]. In addition, HMM alters plant physiological processes, inactivate proteins and disturb the cell membrane [3], which eventually reduce enzyme activities, respiration and metabolism [4–6]. HMMs are considered as one of the major abiotic stresses, which affects soil microbial population and community structure [7,8] particularly arbuscular mycorrhizal fungi (AMF). AMF belong to phylum Glomeromycota [9], which form a symbiotic association with more than 80% of terrestrial plants [10]. Dominant plant species present in HMM contaminated sites are habitually colonized by AMF, which is indicative of their central role in mitigating heavy metal stress in plants [11–13]. AMF perform two different roles in phytoremediation and successful establishment of plants in HMM contaminated sites, one being phytoextraction and another, phytostabilization [14,15].

Janghang smelter (N 36°00’27.92”, E 126°39’59.71”) is located in south Chungcheong province, South Korea. Under operation for more than 50 years, the smelter was closed in 1989 due to metal and metalloid pollution around the smelter [16]. In September 2009, the government decided to buy up the surrounding land, relocating 372 resident households and planned to spend the following few years to carry out soil HMM remediation [17]. The recent report of Tipayno et al. [8] revealed that HMM have negative influences on soil bacterial activity and diversity in and around Janghang smelter. Some recent studies have shown that AMF activity and diversity are influenced by soil heavy metal [18], trace metal [19] and arsenic pollution [20]. The results of these studies showed that community structure and species dominance changes based on the type, concentration and combinations of HMM present in the soil. In addition, they also revealed that *Glomeraceae* is dominate in contaminated sites located in different parts of the world. However, knowledge on AMF diversity and dominant AMF species present in HMM contaminated soils around Janghang smelter remains to be studied which may contribute to better plant growth and phytoremediation process.

In traditional method, AMF spore morphological characters has been considered as an important tool for identification and diversity analysis [21]. However, some AMF species shared similar spore wall characters and spore morphology varies with different stages of spore formation [18,22]. Due to the difficulties in spore morphological study, molecular techniques are widely employed to study AMF community structure, which lead to obtain more precious information than that of traditional method [23]. Terminal restriction fragment length polymorphism (T-RFLP), denaturing gradient gel electrophoresis (DGGE) and pyrosequencing are the widely used fingerprinting techniques to study AMF community structure [19,24–26]. Nonetheless, each fingerprinting techniques have its own pros and cons, use of more than one technique may give reliable information on AMF community structure rather than using single molecular method [27]. In line with this, we have assessed the impact of HMM contaminated soils on AMF abundance and community in different sites using three complementary methods, which are spore morphology, T-RFLP and DGGE.

## Materials and Methods

### Study sites, sampling and soil analysis

Soil samples were collected in and around Janghang smelter (36^o^ 00’ N; 126^o^ 39’ E) located in South Chungcheong province, South Korea. This province receives 1188 mm of annual precipitation and the annual average temperature is 12°C. Soil samples were collected from three different sites located at 240 m (site 1), 260 m (site 2) and 3500 m (site 3) away from the smelter. No special permissions were required to access the sampling locations. The location is not protected in any way and our study did not involve endangered or protected species. The study site was dominated by *Cosmos bipinnatus*, *Hymenachne amplexicaulis*, *Phragmites* sp., *Conyza canadensis* and *Bidens tripartite* plant species. Soil samples were collected at a depth of 0–30 cm from three different locations at varying distances from the refinery center (S1 Fig). Four bulk soil samples were collected from each site by leaving at least 15 m between samples. In each sampling site the samples were collected from 12 sampling points (totally four samples per site; for each sample three subsamples were taken and mixed to make approximately 1 kg of soil) representing all land use area. Collected samples were packed in an icebox and transported to the laboratory. Each soil sample was separately mixed well and divided into two parts, one was placed in -20°C for molecular experiments and the other was stored at room temperature for soil analysis and spore isolation. Soil properties such as EC, pH, and available phosphorus content were determined by standard laboratory protocols. Extractable heavy metal and metalloid (Cd, Cu, Ni, Pb, Zn and As) were analyzed using inductively coupled plasma optical emission spectrometry (ICP-OES) after 0.1/1.0 N HCl extraction.

### Spore isolation and spore morphological study

AMF spores were isolated from 100 g of soil by wet sieving followed by sucrose centrifugation [28,29]. Isolated spores were morphologically grouped into different family based on spore morphological description and classification given by Oehl et al. [30], Kruger et al. [31] and Redecker et al. [32]. Spore density (SD) and family relative abundance (FRA) were calculated as follows: SD was taken as the number of AMF spores per 100 grams of soil; FRA was calculated as number of spores of a given family / total number of spores x 100 (modified from [33]).

### Soil DNA isolation

Total soil DNA of twelve samples were isolated using power soil DNA isolation kit (MO BIO Lab., Inc, USA) following the manufacture’s instruction. Isolated DNA was used as a template for T-RFLP and DGGE analysis.

### T-RFLP and AMF community structure analysis

T-RFLP was performed in twelve samples by targeting larger subunit (LSU) of AMF rDNA with specific primers. Nested PCR was performed with two sets of primers, LR1/FLR2 was used for the first round PCR. The resulting PCR product was diluted 20 folds (using TE buffer) and used as a template for second round PCR with FLR3/FLR4, the size of the PCR product was approximately 380bp [34]. Courtney et al. [35] proved that nested PCR amplification of AMF large subunit with FLR3/FLR4 followed by *Alu*I and *Mbo*I digestion was found to be effective in detecting AMF species present in very low frequency in the soil. FLR3/FLR4 was fluorescently labeled at 5’ end with FAM and HEX, respectively. PCR reaction was comprised of 50 μl reaction mixture containing 1 μl of template DNA, 0.2 mM of each dNTP, 0.4 μM of each primer, 5 μl of 10x Taq buffer and 1.25 units of Taq DNA polymerase. Thermal cycles included one step initial denaturation for 5 min at 95°C, 25 cycles (30 cycles for nested PCR) consisting of 1 min at 95°C, 1 min at 56°C (58°C for nested PCR) and 1 min at 65°C followed by a final extension for 10 min at 65°C. PCR products were purified using a QIAquick PCR purification kit (QIAgen, Germany) with an elution product of 60 μl. Purified PCR products were separately digested with the restriction enzyme *Alu*I and *Mbo*I (Promega, USA). Sixteen microliters of PCR product and five units of restriction enzyme in the manufacture’s recommended buffer were incubated for 4 h at 37°C for digestion. Terminal restriction fragments (T-RFs) of all samples were determined using ABI 3130 DNA sequencer with ROX 500 (Applied Biosystem, USA) as a standard. Genemapper ver 3.7 (Applied Biosystem, USA) was used to identify and quantify the fluorescent labeled T-RFs.

T-RFs peak identified from individual T-RFLP profiles were compiled, arranged and aligned for statistical analysis using RiboSort software [36]. Peak height less than 50 was discarded to exclude background noise. Similarly, T-RFs length smaller than 40 bp and larger than 400 bp was also excluded. Total average abundance was calculated by dividing the total peak height of a sample with the total number of ribotypes obtained in a particular sample [37]. Richness (S), shannon diversity index (H’) and evenness (J’) was calculated as follows; S was determined by the presence or absence of bands in the electrogram. H’ and J’ was calculated using -∑ (Pi) (*In* pi), and H’/In (S) formulas respectively. Pi, *In* and S in the formula represents the abundance of T-RFs, natural log and the number of T-RFs respectively. Non-metric multidimensional scaling (NMDS) was performed using primer V.6 software package (Prime-E Ltd., Plymouth, UK). Bray Curtis coefficient after presence/absence transformation was performed before calculating similarity matrix. 2D stress was calculated using Kruskal’s stress formula: Stress = √∑_h, i_ (d_hi_-d’_hi_)^2^/∑ _h_, _i_d^2^_hi_ where, d_hi_ is the ordinate distance between samples h, i, and d’ are the distance predicted from the regression. Based on the bands in DGGE presences/absence matrix was built for statistical analysis. Richness and shannon diversity index (H’) for DGGE was calculated as mentioned above.

Web based Microbial community analysis (MiCA) tool (http://mica.ibest.uidaho.edu/) was used to convert the T-RFs into possible ribotypes [38]. T-RFLP phylogenetic assignment tool (PAT+) option was used in MiCA [39]. Primers, restriction enzymes and data file (abundance and fragment size) were fed into the tool. Then, SILVA (R106) 207, 909 LSU (23/28S) Parc database was used with zero mismatches in digest and ±1bp bin tolerance for all fragment lengths.

### DGGE and *Glomeraceae* community structure analysis

Total DNA was subjected to three subsequent PCRs, first PCR was performed with GeoA2 and Geo11 primers [40] which targets fungal DNA from total DNA. The second PCR primers NS31 and AM1 [41] amplified 550 bp of AMF and third PCR was performed with NS31-GC and Glo1 [42] primers which amplifies approximately 250 bp of *Glomeraceae* DNA. PCR products were diluted 40 folds (using TE buffer) before performing the next PCR. PCR reactions were performed in a final volume of 20 μl containing 2 μl of 10X PCR buffer (containing Mg^+^), 400 μM dNTP mix, 0.2 μM primers, 1 unit of Taq DNA polymerase and 2 μl of template DNA. Thermo cycle conditions for the first PCR was performed as follows, initial denaturation at 94°C for 4 min; 30 cycles of 94°C for 1 min; 54°C for 1 min; 72°C for 2 min and a final extension at 72°C for 7 min. The second PCR was performed with one cycle of 94°C for 1 min; 66°C for 1 min; 72°C for 1.30 min; 30 cycles of 94°C for 30 s; 66°C for 1 min; 72°C for 1 min and a final extension at 72°C for 10 min. Third PCR was performed with an initial denaturation at 95°C for 5 min; 35 cycles of 94°C for 45 s; 52°C for 45 s; 72°C for 1 min and a final extension for 30 min at 72°C. PCR products were purified and used for DGGE analysis.

DGGE was performed by using 15 μl of PCR product in 8.0% polyacrylamide gel with 35 to 55% denaturant containing 7M urea and 40% formamide. Initial electrophoresis was performed with 75V for 20 min and increased to 120V for 10 h at a constant temperature of 60^°^C. After electrophoresis, the gel was stained in 1X TAE buffer containing 0.5 μg of ethidium bromide per ml and destained using the same buffer without ethidium bromide [43]. The bands in DGGE gel were excised using scalpel and eluted in 20μl of sterile distilled water by incubating at 4°C for overnight [44]. Eluted DGGE bands were diluted 40 folds and used as a template (2.0μl) for PCR reaction (NS31-GC/Glo1 primers) with the same conditions as described above in order to test the existence of double bands. The PCR products were sequenced using NS31/Glo1 primers in automated ABI 3100 sequencer (Solgent, South Korea). Nucleotide sequences data obtained in this study was compared with those from the genbank (http://www.ncbi.nlm.nih.gov/genbank/) using the BlastN program [45]. The sequences were submitted in genbank under the accession number of KC887744-KC887757. 18S rDNA sequences were aligned using the CLUSTAL W program and phylogenetic tree was constructed using MEGA version 5.2 software package. Neighbor-joining tree was constructed with bootstrap values of 1000.

### Statistical analysis

Pearson correlation coefficient analysis was performed using SPSS software ver. 19 to find out the relationship between HMM and AMF diversity. Data on spore count and molecular studies were subjected to analysis of variance (ANOVA). Significance at the level of 5.0% was tested by *t* test using the SAS package, version 9.1.3. Linear regression analysis was performed to examine the relationship between soil HMM (As, Cd and Zn) and *Glomeraceae* spore count, richness and diversity.

## Results

### Soil HMM concentration and spore morphological study

**Fig 1:**
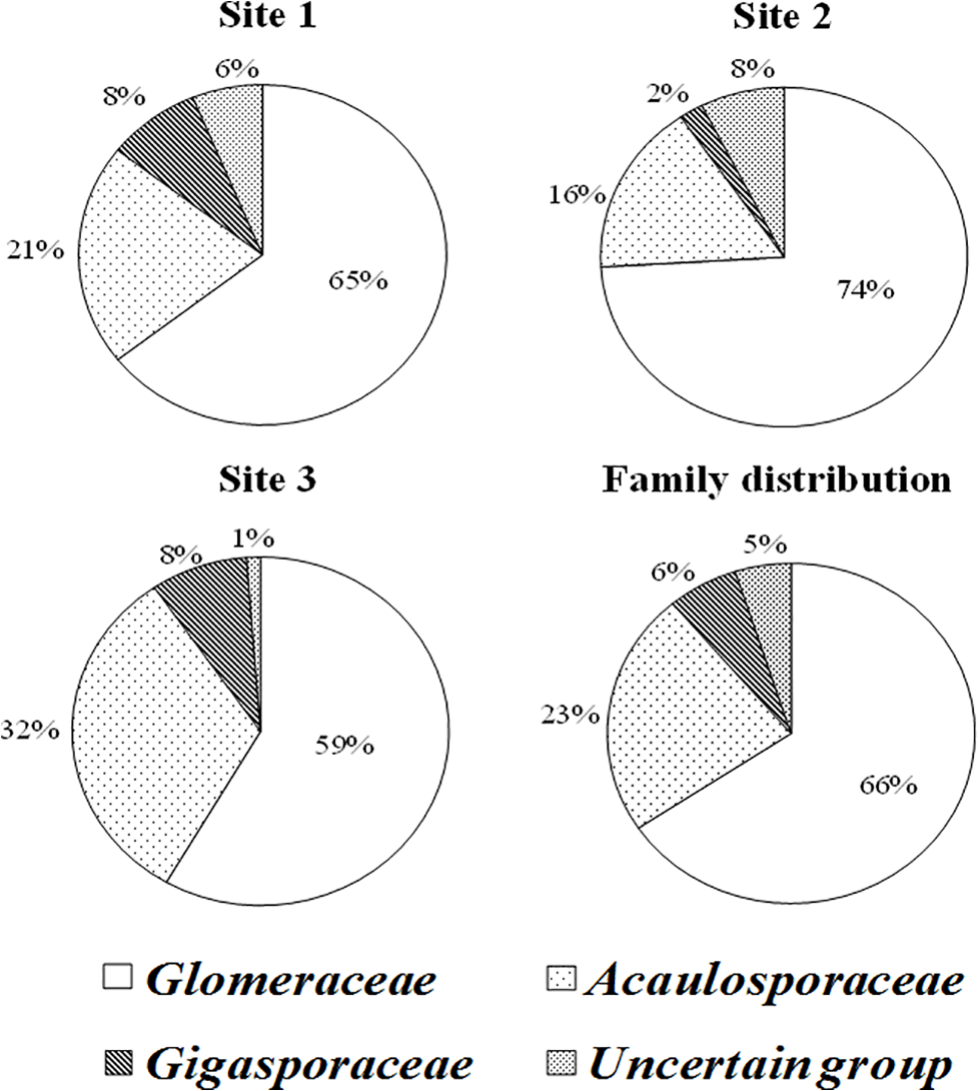
Family relative abundance (FRA) of arbuscular mycorrhizal fungi in heavy metal contaminated soils.

Soil chemical properties of HMM contaminated sites are given in Table 1. Based on soil HMM concentration (except Ni) site 1, 2 and 3 may be considered as highly, moderately and less contaminated sites, respectively. HMM concentration of site 1 was significantly higher than site 3. Spore density was significantly higher in site 1 compared to other two sites (S2 Fig). Among different AMF families, *Glomeraceae* was found to be dominant in HMM contaminated soils, followed by *Acaulosporaceae*, *Gigasporaceae* and uncertain group (= *Entrophosporaceae*). The average family relative abundance in all three sites was higher for *Glomeraceae* (66%) followed by *Acaulosporaceae* (23%) and others contributed 11 percent (Fig 1).

**Table 1:** Soil chemical properties of samples collected from HMM contaminated sites.

Samples	pH	EC_se_	Av.P_2_O_5_	As	Cd	Cu	Ni	Pb	Zn
	(1:5)	dS m^-1^	mg kg^-1^ of soil						
**Site 1**	**7.0±0.02** ^**a**^	**0.2±0.02** ^**a**^	**1436±198** ^**a**^	**17.9±1.8** ^**a**^	**3.1±0.2** ^**a**^	**122.9±4.2** ^**a**^	**4.4±0.2** ^**ab**^	**219.9±4.1** ^**a**^	**67.3±8.1** ^**a**^
**Site 2**	**6.2±0.15** ^**b**^	**0.3±0.06** ^**a**^	**943±482** ^**a**^	**9.3±4.2** ^**ab**^	**2.2±0.2** ^**b**^	**118.5±4.8** ^**a**^	**4.0±0.0** ^**b**^	**179.1±34.7** ^**a**^	**25.9±5.3** ^**b**^
**Site 3**	**6.6±0.05** ^**ab**^	**0.2±0.04** ^**a**^	**967±27** ^**a**^	**4.9±0.1** ^**b**^	**1.1±0.0** ^**c**^	**21.8±2.8** ^**b**^	**4.6±0.1** ^**a**^	**14.9±2.1** ^**b**^	**17.3±3.4** ^**b**^

Values presented here are the mean of four replicas ± SE (standard error). Same letters in the column are not significantly different between sites at *P* < 0.05.

### AMF community structure analysis by T-RFLP

Number of T-RFs obtained from enzymatic digestion was found to be higher in site one compared to other two sites. Twenty one percent of T-RFs obtained from *Alu*I and *Mbo*I digestion were found to be present in all three sites (S3 Fig). In addition, six and twenty percent of unique T-RFs originated from site 2 and site 3, respectively. Fifteen percent of fragments were observed only in site one and the remaining were inconsistent among different sites. Total average abundance of AMF was found to be significantly higher in site 1 compared to site 3 (S4 Fig). NMDS analysis results revealed that AMF species present in site 1 tend to group together, whereas site 2 and site 3 samples exhibited a distinct relationship with one another. *Alu*I digestion showed that AMF species present in site 1 significantly differed from site 3 (S5 Fig). Diversity indices estimated using shannon formula and evenness were not significantly different with the levels of contamination.

**Fig 2:**
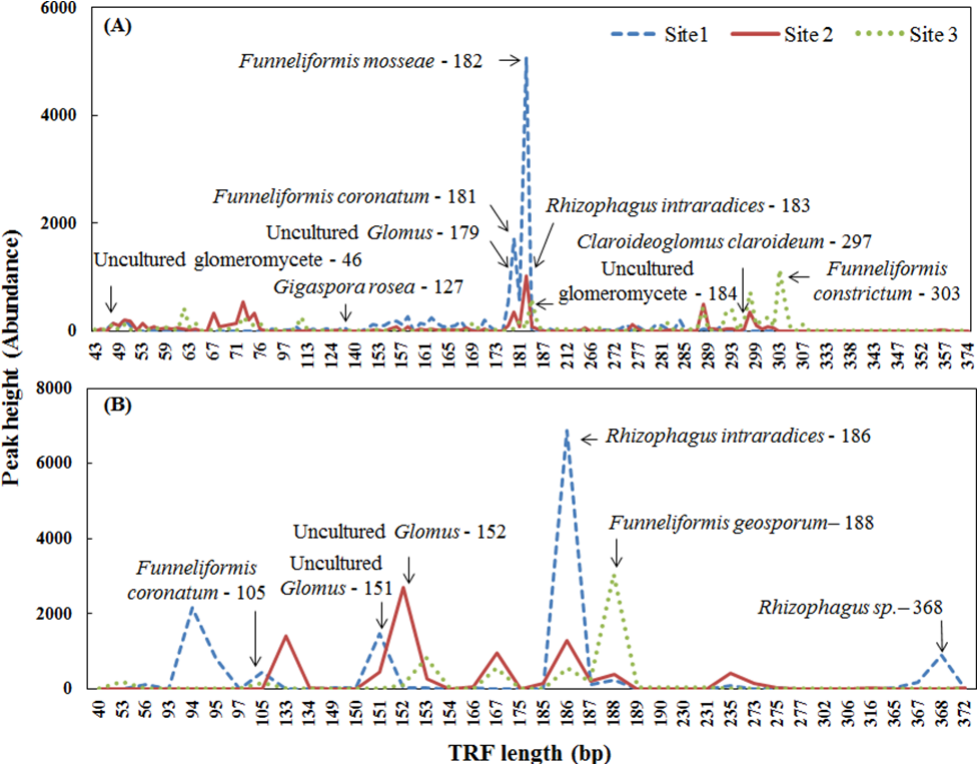
Peak abundance of Terminal Restriction Fragments (T-RFs) and AMF ribotype corresponds to fragment size. (A) *Alu*I, (B) *Mbo*I digestion.

Abundance of various terminal restriction fragments from 40 to 400 bp is presented in Fig 2. Possible species name for the 27 ribotypes of T-RFs obtained from *Alu*I and *Mbo*I digestion by using MiCA tool is reported in S1 Table. Site 1 was found to be dominated by *Funneliformis mosseae*, *Funneliformis coronatum*, *Rhizophagus intraradices*, uncultured *Glomus* and *Rhizophagus* sp. Similarly, site 2 was dominated by *F*. *mosseae*, *R*. *intraradices* and uncultured *Glomus*. *Funneliformis constrictum*, *Claroideoglomus claroideum*, *R*. *intraradices* and *Funneliformis geosporum* were dominant in site 3 (Fig 2).

### *Glomeraceae* diversity analysis by DGGE

**Fig 3:**
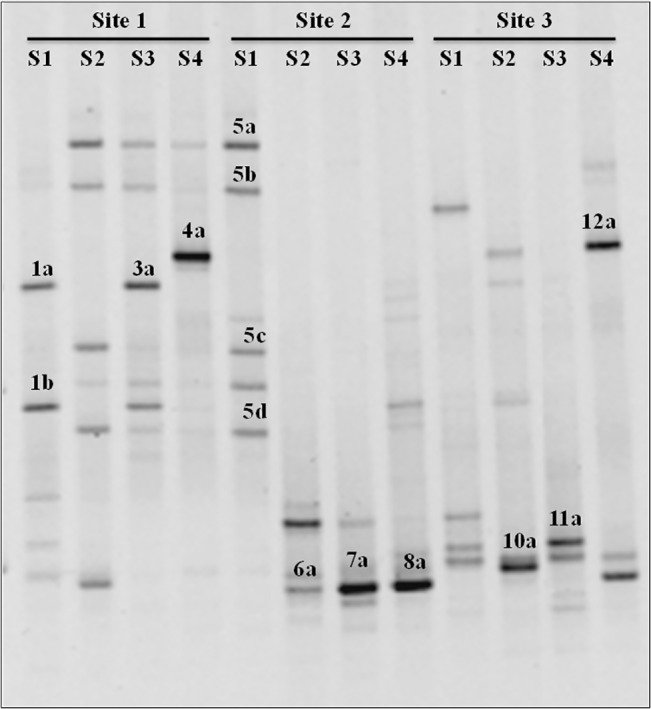
Denaturing Gradient Gel Electrophoresis (DGGE) profile of partial 18S rDNA amplification of *Glomeraceae* family. S1-S4 represents the samples obtained from each site. Band numbering refers to *Glomeraceae* ribotypes identified by sequencing are given in fig 4.

**Fig 4:**
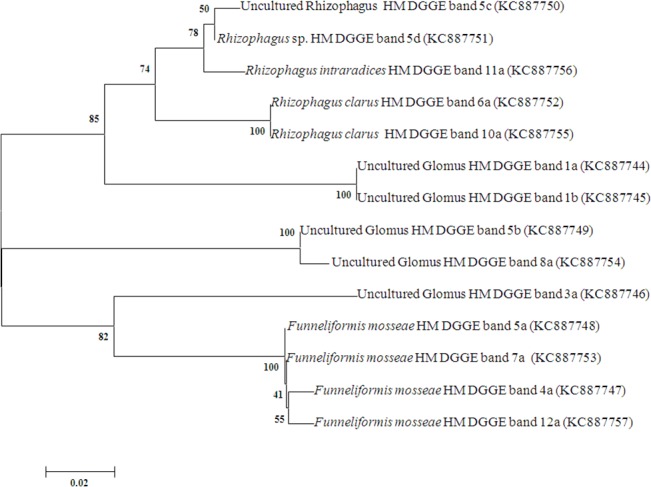
Nearest BLAST match name of the dominant *Glomeraceae* sequences obtained from DGGE gel.

DGGE profile of *Glomeraceae* from different HMM contaminated soils was shown in Fig 3. The sequence of dominant bands obtained from DGGE analysis and their nearest neighbor from genbank were presented in Fig 4. All three sites were dominated by uncultured *Glomus* and *F*. *mosseae*. In addition, site 2 and site 3 were dominated by *R*. *clarus* and *R*. *intraradices*. DGGE analysis and sequencing result revealed that uncultured *Glomus*, *R*. *clarus*, *R*. *intraradices* and *F*. *mosseae* were dominant in HMM contaminated sites. Richness and shannon diversity index of *Glomeraceae* were found to be significantly higher in site 1 compared to the other two sites (S6 Fig).

### Correlation analysis

**Fig 5:**
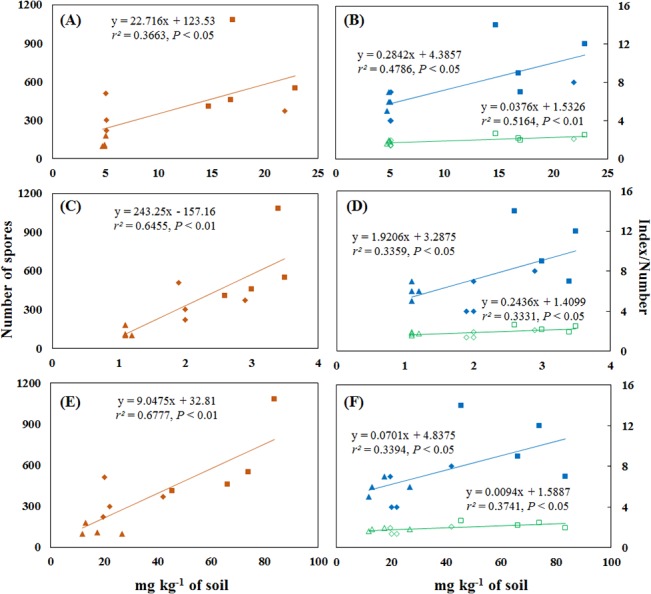
Regression analysis of soil As (A, B), Cd (C, D) and Zn (E, F) against *Glomeraceae* spore count (A, C, E), richness and diversity (B, D, F). Square, diamond and triangle represents site 1, site 2 and site 3 respectively. In Fig 5 B,D and F the open data series represents the diversity index and the closed ones are represents the richness.

Soil HMM concentration with the exception for Ni showed significant positive correlation to total spore count (*P* < 0.05), *Glomeraceae* (*P* < 0.05) and uncertain group (*P* < 0.05) spore count. *Acaulosporaceae* spore count was found to be positively correlated with As (*P* < 0.01), Cd (*P* < 0.01), Pb (*P* < 0.05) and Zn (*P* < 0.01). Soil As, Zn and Cd concentrations exhibited a significant positive correlation for *Gigasporaceae* (*P* < 0.05) spore count, and total average abundance (*P* < 0.05) was positively correlated to soil Cd and Zn (Table 2). In addition, As, Cd and Zn exhibited a significant positive correlation to *Glomeraceae* spore count (*P* < 0.05), species richness (*P* < 0.05) and shannon diversity index (*P* < 0.05) analyzed by DGGE (Fig 5).

**Table 2:** Pearson correlation coefficient between soil heavy metals and metalloid, spore count, T-RFLP and DGGE of arbuscular mycorrhizal fungi.

	Spore morphology					T-RFLP				DGGE	
	SD	G.	A.	G.	U.	TAA	S	H’	J’	S	H’
**As**	0.724**	0.605*	0.914**	0.596*	.689*	**0.502**	**0.231**	**0.065**	**-0.166**	0.692*	0.719**
**Cd**	0.869**	0.803**	0.841**	0.698*	.843**	0.644*	**0.491**	**0.398**	**0.131**	0.580*	0.577*
**Cu**	0.694*	0.695*	**0.525**	**0.422**	.753**	**0.541**	**0.499**	**0.448**	**0.228**	**0.370**	**0.329**
**Ni**	**0.251**	**0.183**	**0.374**	**0.404**	**.103**	**0.218**	**0.056**	**0.031**	**-0.024**	**0.055**	**0.145**
**Pb**	0.745**	0.704*	0.688*	**0.506**	.790**	**0.551**	**0.408**	**0.295**	**0.031**	**0.520**	**0.506**
**Zn**	0.912**	0.823**	0.914**	.885**	.813**	0.700*	**0.482**	**0.397**	**0.086**	0.583*	0.612*

*Correlation is significant at the 0.05 level

**Correlation is significant at the 0.01 level

SD—Spore density; G.—*Glomeraceae*; A.—*Acaulosporaceae*; Gi.—*Gigasporaceae*; U.—Uncertain group; TAA—Total average abundance; S—Richness; H’—Shannon diversity index; J’—Evenness.

## Discussion

Soils adjacent to Janghang smelter was found to contain high concentrations of extractable As, Zn, Cd, Cu and Pb compared to the sampling site located at 3500 m away from the smelter. Based on the soil HMM criteria set by the Korean Ministry of Environment, site 1 is considered as highly contaminated and remediation strategies should be applied in order to use the land for agricultural purposes [46]. Although HMM concentration in site 1 is very high, care should be taken when interpreting these results because the critical level of soil HMM concentration set by governments varies among different countries. Soil HMM contamination causes toxic effects to plants, reduces nutrient availability and inhibits plant growth [47]. Root colonization by AMF has been found to enhance plant establishment in contaminated soils and colonization has positive correlation with plant growth [12]. AMF symbiosis supports plant heavy metal stress alleviation through processes such as phytoextraction or phytostabilization [15,48].

Higher root colonization of plants in contaminated soil enhances production of hyphae that are extraradical (EH) in soil. When these EH are exposed to soil HMM, AMF triggers the formation of spore producing hypha as a survival strategy, which leads to higher spore production. In line with this, root colonization and spore density were found to be higher in contaminated soil compared to non contaminated soils of India [49] and in the vicinity of a smelter in France [50]. Helgason and Fitter [51] reported that rapid production of spore was likely to be an adaptation mechanism for AMF survival under stress condition. In agreement with this, our result also showed significantly higher spore density in highly contaminated soil than that of the less contaminated soil. *In vitro* study with *R*. *intraradices* showed that EH and spore density in root compartment increased with increase in Pb, Zn and Cd concentration [52]. Similarly, our study also showed the increase in spore density with relation to soil As, Cd, Cu, Pb and Zn concentration. Moreover, Zarei et al. [11] also reported a positive correlation between spore density and soil Zn concentration.

*Glomeraceae* family has more adaptability and adjusting pattern of sporulation under stress conditions than other families of AMF [53]. Our spore morphological study result support the previous finding where *Glomeraceae* was found to be well adapted to HMM contaminate soil [19]. In addition, relative abundance of *Glomeraceae* was found to be higher in all three study sites used in the present work. Gonzalez-Chavez et al. [54] and Schneider et al. [20] also reported the dominance of *Glomeraceae* in an arsenic mining site. Significant increase in the *Glomeraceae* spore count was observed with increasing concentrations of soil As, Cd, Cu, Pb and Zn. These results support our hypothesis that *Glomeraceae* species could be the dominant AMF population in the vicinity of an abundant Janghang smelter. Recently Aguilera et al. [55] and Pereira et al. [56] used spore wall characters, subtending hypha and germination structure for species level diversity analysis. However, in our spore morphological study, we have used spore wall characters, subtending hypha and germination structure only to analyze family level diversity due to the difficulties in genus/species level differentiation. The nomenclatures and classification of AMF followed in this work was based on Schußler and Walker [57] and Redecker et al. [32]. In our spore morphological study, the spores belongs to *Entrophosporaceae* family are denoted as uncertain group as mentioned by Redecker et al. [32].

Community structure analysis by T-RFLP revealed that AMF species present in soil vary with respect to soil HMM concentration. Further, MiCA analysis confirmed the presence of *F*. *mosseae*, *F*. *coronatum*, *F*. *geosporum* and *R*. *intraradices* in all three sites. These AMF species might have developed adaptation and tolerance mechanism(s) to different levels of soil HMM concentration. Metal tolerant AMF species could germinate and colonize plant roots even at high Pb, Cd and Zn concentration in soil and improve plant tolerance against metal stress [52]. Experimental evidences showed that metal tolerant *F*. *mosseae* improved *Medicago truncatula Gaertn*. growth in soils with high concentration of As (200 mg/kg of soil) [48]. Our result supported the previous findings of Ortega-Larrocea et al. [58] where *F*. *mosseae* and *R*. *intraradices* were dominant in heavy metal contaminated soils of Mexico. These two AMF species were also found to be dominant in phosphate [59], Pb and Zn [60], trace metals [19] and As contaminated soils [20]. However, *R*. *intraradices* was totally absent in zinc and lead mining regions [11]. The results from the above mentioned studies indicates that the abundance of particular AMF species might be influenced by the type and amount of HMM present in the soil.

Interestingly, in our study, 12% of unique T-RFs were found only in highly contaminated sites. Among these, two were identified as uncultured Glomeromycota and *Gigaspora rosea*. Heavy metals were deposited on the soils surrounding the smelter area for more than 50 years which led AMF species present in that soil to develop adaptation mechanisms against HMM stress. Selective pressure of heavy metals on AMF species present in highly contaminated soil may have altered the species richness and led to enrichment with particular species over a period. Absence of these AMF ribotypes in moderately and less contaminated sites may be due to the dominance of other species. Forty six percent of unique T-RFs were absent in highly contaminated soil, while they were present only in moderately and less contaminated soils. These species may be sensitive to elevated level of HMM and were identified as *Claroideoglomus claroideum*, *F*. *caledonium*, *R*. *clarus* and *F*. *constrictum*. Zarei et al. [60] previously documented the heavy metal sensitive nature of *F*. *claroideum*. Based on the presence and absence of T-RFs in three different contaminated soils, AMF species could be grouped under three possible categories. First group consists of tolerant strains that are found only in highly contaminated site. Secondly, strains that showed high levels of adaptability (*F*. *mosseae*, *F*. *coronatum*, *F*. *geosporum* and *R*. *intraradices*), which were present in all three sites. The final group consisted of sensitive strains, which were observed only in the less contaminated site.

Total average abundance (population) of AMF was found to be significantly higher in highly contaminated soil followed by moderately and less contaminated soils. AMF population showed significant positive correlation for soil HMM, particularly soil Cd and Zn. Positive correlation between total average abundance by T-RFLP and spore count was more apparent in highly contaminated soil than in less contaminated sites. Our NMDS analysis result revealed that selective pressure of HMM in highly contaminated soil leads to favor the growth of similar AMF species. Kruska’s stress value obtained in NMDS was lower than 0.06 which, indicating a good representation of the relationship between points in the matrix. Even though AMF species distribution varies among different levels of contamination, richness and diversity indices were not significantly different. Other authors have also reported similar results where species richness and diversity indices did not significantly vary with increasing HMM concentrations [19,21]. Contradictory to this, Wu et al. [61] reported a significantly higher AMF diversity in HMM contaminated sites compared to non contaminated sites.

Sequencing of dominant DGGE bands confirmed the results obtained in T-RFLP where *F*. *mosseae* and uncultured *Glomus* were dominant in all three sites. In contradiction to our T-RFLP results, dominant bands sequenced from DGGE revealed the presences of *R*. *intraradices* only in low contaminated soils; this may be due to less number of bands sequenced. *Glomeraceae* richness and shannon diversity indices increased as soil As, Cd and Zn concentration increased. In line with this, many authors reported *Glomeraceae* dominance in heavy metal contaminated sites [59,60,62].

As can be inferred from our spore morphological, T-RFLP and DGGE results, a polyphasic approach rather than using a single technique to study AMF community structure may give us reliable information on AMF community structure and dominant species present in contaminated sites. In addition, these analyses confirmed the presence and dominance of *F*. *mosseae* and *R*. *intraradices* in all sites irrespective of HMM concentration. *Glomeraceae* spore count, richness and diversity improved with significant increase in soil As, Cd and Zn concentrations. Therefore, understanding the nature of As, Cd and Zn tolerance, and uptake or biotransformation by native dominant *Glomeraceae* genus/species would be valuable in plant growth promotion or phytoremediation process in the HMM contaminated sites.

## Supporting Information

S1 Fig Sampling locations of heavy metal and metalloid contaminated sites in the vicinity of Janghang smelter. (TIF)

S2 Fig Spore density and total spore count of AMF families in heavy metal contaminated sites. Data are presented as mean ± SE (standard error) from four replications; letters shows significant differences between sites according to t—test (*P* < 0.05).
(TIF)

S3 Fig Graphical representation of Terminal Restriction Fragments (T-RFs) occurrence in three different contaminated sites. T-RFs from *Alu*I (A) and *Mbo*I (B) digestion of Large Sub Unit. Horizontal bars represent the presences of the particular bp in the particular site and absences of bars indicate that the particular bp is not found in that particular site.
(TIF)

S4 Fig Total average abundance of AMF in different contaminated soils (average obtained from *Alu*I and *Mbo*I digestion). Data are presented as mean ± SE from four replications; letters shows significant differences between sites according to t—test (*P* < 0.05).
(TIF)

S5 Fig Non-metric multidimensional scaling (NMDS) plot based on Bray—Curtis similarities of AMF community from T-RFLP analysis. Points represent AMF species associated with highly (open circle), moderately (open square) and less contaminated soil (closed square). (A) *Alu*I, (B) *Mbo*I digestion.
(TIF)

S6 Fig Richness (A) and Shannon diversity index (B) of *Glomeraceae* present in different heavy metal contaminated sites. Data are presented as mean ± SE from four replications; letters shows significant differences between sites according to t—test (*P* < 0.05).
(TIF)

S1 Table Terminal restriction fragment size from *Alu*I and *Mbo*I digestion and respective ribotype obtained from MiCA. (DOCX)
