# Supplementary figures and images for: Arbuscular Mycorrhizal Fungi Community Structure, Abundance and Species Richness Changes in Soil by Different Levels of Heavy Metal and Metalloid Concentration
Source: PLoS One. 2015 Jun 2;10(6):e0128784. doi: 10.1371/journal.pone.0128784 (PMC4452772; doi:10.1371/journal.pone.0128784)

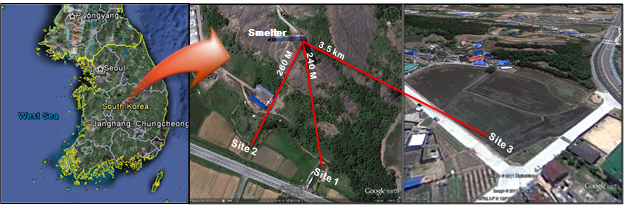

Supplement: S1 Fig — (TIF) [file pone.0128784.s001.tif]

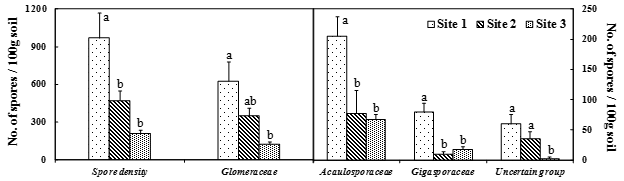

Supplement: S2 Fig — Data are presented as mean ± SE (standard error) from four replications; letters shows significant differences between sites according to t—test (P < 0.05). (TIF) [file pone.0128784.s002.tif]

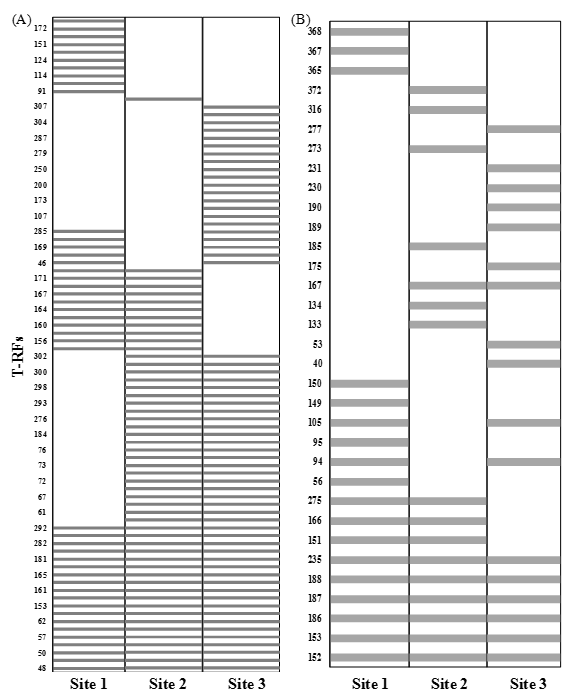

Supplement: S3 Fig — T-RFs from AluI (A) and MboI (B) digestion of Large Sub Unit. Horizontal bars represent the presences of the particular bp in the particular site and absences of bars indicate that the particular bp is not found in that particular site. (TIF) [file pone.0128784.s003.tif]

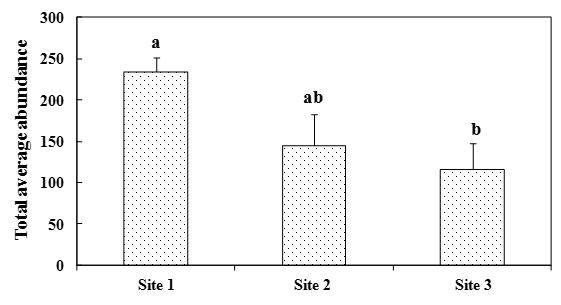

Supplement: S4 Fig — Data are presented as mean ± SE from four replications; letters shows significant differences between sites according to t—test (P < 0.05). (TIF) [file pone.0128784.s004.tif]

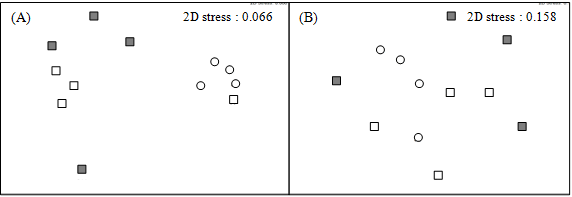

Supplement: S5 Fig — Points represent AMF species associated with highly (open circle), moderately (open square) and less contaminated soil (closed square). (A) AluI, (B) MboI digestion. (TIF) [file pone.0128784.s005.tif]

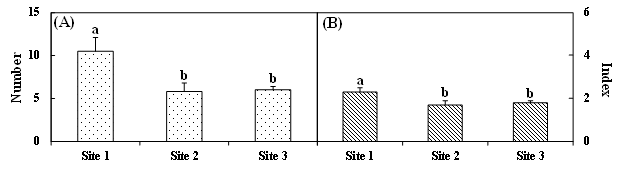

Supplement: S6 Fig — Data are presented as mean ± SE from four replications; letters shows significant differences between sites according to t—test (P < 0.05). (TIF) [file pone.0128784.s006.tif]
