# Supplementary material for: Arbuscular Mycorrhizal Fungi Community Structure, Abundance and Species Richness Changes in Soil by Different Levels of Heavy Metal and Metalloid Concentration
Source: PLoS One. 2015 Jun 2;10(6):e0128784. doi: 10.1371/journal.pone.0128784 (PMC4452772; doi:10.1371/journal.pone.0128784)
Supplement: S1 Table — (DOCX) [file pone.0128784.s007.docx]

**Table S1** **Terminal restriction fragment size from *Alu*I and *Mbo*I digestion and respective ribotype obtained from MiCA.**

| **T-RF size (bp)** | **Possible ribotype** | **Accession number** |
| --- | --- | --- |
| ***Alu*I digestion** | | |
| 46 | Uncultured glomeromycete | GQ149168 |
| 127 | *Gigaspora rosea* | AM040348 |
| 170 | Uncultured glomeromycete | GQ149221 |
| 171 | Uncultured glomeromycete | GQ149221 |
| 179 | Uncultured *Glomus* | AB369766 |
| 180 | *Rhizophagus intraradices* | FM865565 |
| 181 | *Funneliformis coronatum* | AF304881 |
| 182 | *Funneliformis* *mosseae* | AY639276 |
| 183 | *Rhizophagus* *intraradices* | FM865598 |
| 184 | Uncultured glomeromycete | GQ149156 |
| 297 | *Claroideoglomus claroideum* | AY639331 |
| 298 | *Claroideoglomus claroideum* | AY639184 |
| 300 | *Claroideoglomus claroideum* | AY639188 |
| 301 | Uncultured glomeromycete | GQ149154 |
| 302 | *Rhizophagus clarus* | AJ510243 |
| 303 | *Funneliformis constrictum* | AF304977 |
| 304 | *Funneliformis coronatum* | FM876798 |
| 307 | *Funneliformis caledonium* | AF396789 |
| ***Mbo*I digestion** | | |
| 105 | *Funneliformis* *coronatum* | AF304946 |
| 149 | Uncultured glomeromycete | GQ149209 |
| 150 | Uncultured glomeromycete | GQ149224 |
| 151 | Uncultured *Glomus* | AB561094 |
| 152 | Uncultured *Glomus* | AB369738 |
| 186 | *Rhizophagus intraradices* | AY541855 |
| 188 | *Funneliformis* *geosporum* | AF145742 |
| 367 | Uncultured *Glomus* | AB561128 |
| 368 | *Rhizophagus* sp. | AM040332 |
